# Climacteric symptoms in postoperative patients among endometrial cancer, cervical cancer, and ovarian cancer: a cross-sectional study

**DOI:** 10.1007/s00520-022-07117-z

**Published:** 2022-05-08

**Authors:** Yuko Horiba, Tetsuhiro Yoshino, Megumi Yokota, Takashi Iwata, Kenji Watanabe, Masaru Mimura, Daisuke Aoki

**Affiliations:** 1 Department of Obstetrics and Gynecology, Keio University School of Medicine, 35 Shinanomachi, Shinjuku-ku, Tokyo, 160-8582 Japan; 2 Center for Kampo Medicine, Keio University School of Medicine, 35 Shinanomachi, Shinjuku-ku, Tokyo, 160-8582 Japan; 3 Department of Neuropsychiatry, Keio University School of Medicine, 35 Shinanomachi, Shinjuku-ku, Tokyo, 160-8582 Japan

**Keywords:** Adnexectomy, Climacteric, Gynecologic cancer survivors, Health-related quality of life, Hysterectomy, Patient-reported outcome, Postoperative period

## Abstract

**Purpose:**

To date, no studies have assessed climacteric symptoms after hystero-adnexectomy for endometrial, cervical, or ovarian cancer. Thus, this study aimed to compare climacteric symptoms among patients who underwent surgery for these three cancer types.

**Methods:**

In this cross-sectional study, we interviewed patients who were registered at a menopausal outpatient clinic between January 1999 and July 2016 after undergoing total hysterectomy, intrapelvic only or intrapelvic plus para-aortic lymph node dissection, and bilateral adnexectomy performed via laparotomy as a cancer treatment. Climacteric symptoms were assessed using a patient-reported questionnaire covering core domains with five symptoms only at the initial consultation. Each symptom was graded from 0 (no symptoms) to 3 (severe symptoms). We evaluated the frequency of symptom severity according to the time elapsed since surgery and the cancer type.

**Results:**

The numbers of patients with endometrial, ovarian, and cervical cancer were 328, 90, and 107, respectively. Overall, climacteric symptoms were more severe in patients with cervical cancer than in those with endometrial or ovarian cancer; symptom severity decreased with increasing time since surgery. However, symptom severity did not decrease significantly over time in patients with cervical cancer even after > 5 years had elapsed since surgery.

**Conclusion:**

The climacteric symptoms were less severe in patients with endometrial or ovarian cancer with longer time elapsed since surgery but not in those with cervical cancer. Patients with cervical cancer may require more prompt interventions, including symptomatic treatment and longer follow-up period, than those with endometrial or ovarian cancer.

**Supplementary Information:**

The online version contains supplementary material available at 10.1007/s00520-022-07117-z.

## Background

Gynecological cancer has major societal and economic implications, partly because of cancer treatment–related infertility and the role of women in the workforce. Patients with gynecologic cancer are at a higher risk of employment disruption than non-cancer controls [1]. The annual number of women with endometrial, ovarian, and cervical cancers worldwide is 417,367, 313,959, and 604,127, respectively. These cancers were the 15th, 18th, and 7th most common cancer types in 2021, respectively [2]. The 5-year survival rate of patients with gynecological cancer is relatively higher than that of patients with other cancer types [3, 4]; hence, there are many gynecologic cancer survivors.

Long-term gynecologic cancer survivors have significant mental or psychological symptoms [5, 6], but usage of only overall scales of quality of life (QOL), including socioeconomic and family domains in addition to the health-related domain, would be inappropriate to evaluate the survivors’ status or satisfaction [7, 8]. Therefore, further studies focusing on specific symptoms for gynecologic cancer survivors, rather than the overall QOL score, are needed. Treatment of gynecological cancer via uterine and/or ovarian resection is considered to have marked physical and mental effects that are specific to gynecological cancer, including loss of sense of femininity and bilateral adnexectomy. Especially, removing ovaries as an estrogen source results in artificial menopause, which may result in climacteric symptoms for premenopausal patients [9, 10]. For example, ovarian cancer survivors report good overall QOL scores but have impaired sexual function and climacteric symptoms [11]. Gynecological cancer tends to affect women in their 40 s and 50 s [12, 13], ages at which many women experience menopause [14, 15]. Considering the long-life expectancy of such cancer survivors after bilateral adnexectomy, climacteric symptoms may play a role in their life after treatment [16].

Previous studies have reported improvements in QOL and mood [17] or global health status [18] of patients with gynecological cancer with the time elapsed since the treatment. However, the treatment method, cancer stage, and time since cancer diagnosis are not correlated with QOL or mood [19]. Moreover, patients with cervical cancer have lower physical and mental health-related QOL than adults with no cancer history, similar to those of patients with short-term survival cancers (e.g., esophagus, liver, lung, pancreas, and stomach cancers) [20]. Especially, they have worse anxiety, depression, anger, and confusion levels than those with endometrial cancer [19], and their QOL does not reach that of healthy individuals, not even at 2 years postoperatively [21]. Another study showed that more than half of gynecologic cancer survivors have sexual health concerns [22], especially those surviving from cervical cancer [23].

These results are indicative of the need to evaluate differences among these gynecological cancer types, namely, cervical, ovarian, and endometrial cancers. Although it is considered a type of gynecological cancer, cervical cancer is physiologically different as an infectious disease caused by the human papillomavirus, whereas endometrial and part of ovarian cancers are estrogen-dependent. Therefore, the type of gynecological cancer may have a major effect on these differences in addition to the time elapsed since surgery. Therefore, the impact of removing bilateral ovaries as estrogen sources may differ among gynecological cancers [24]. By enrolling patients after bilateral adnexectomy, we can compare patients without adjusting for residual estrogen even after natural menopause [25–27] or operative methods as a possible confounder on post-operation symptoms; however, adjustment for patients’ age remains necessary [28].

Understanding the risk factors associated with worse climacteric symptoms will help identify these individuals and aid in planning interventions for endometrial, ovarian, and cervical cancers. However, there have been no reports, to date, on climacteric symptoms after hystero-adnexectomy for these three cancer types. This study aimed to determine possible risk factors associated with worse climacteric symptoms among patients who underwent endometrial, cervical, or ovarian cancer surgery in an outpatient clinic.

## Methods

### Study setting and patients

This study included patients who were treated at a menopausal outpatient clinic between January 1999 and July 2016. The inclusion criteria were as follows: (i) a histological diagnosis of endometrial, ovarian, or cervical cancer; (ii) postmenopausal state at each first consultation due to total hysterectomy, intrapelvic only or intrapelvic plus para-aortic lymph node dissection, and bilateral adnexectomy performed by laparotomy as cancer treatment; and, if applicable, (iii) completed adjuvant therapy, such as chemotherapy or radiotherapy, at the first consultation, even after recurrence.

Patients were excluded from the analysis if their data were missing (i.e., if the body mass index [BMI] was not recorded) and/or if one or more of the following information was unknown: date of surgery; performance or non-performance and details of adjuvant therapy (chemotherapy and/or radiotherapy); and pre- or postmenopausal status at the surgery.

Details of the following demographic characteristics of the study patients were obtained from medical records: age at first consultation at the menopausal outpatient clinic, cancer type, age at surgery, time since surgery, BMI, menopausal status at surgery, and whether or not chemotherapy and radiotherapy were performed.

### Questionnaire

At the initial examination, the participants were interviewed using a patient-reported questionnaire comprising 40 questions (Online Resource 1), which was prepared based on Kupperman’s menopausal index that was developed in the mid-twentieth century [29–31]; our registry was developed in the 1990s when this index was still being widely used. However, Kupperman’s index has been heavily criticized; therefore, we hesitated to analyze the total score of the 40-question questionnaire [32], and rather than summing up the 40 items, we focused on the core menopausal symptoms, namely, two from vasomotor (hot flashes and sweats) [33] and insomnia (difficulty falling asleep and arousal during sleep) [34], and one vaginal symptom (i.e., vaginal dryness) [35, 36].

Each of the patients’ symptoms was graded on a 4-point scale as follows: 0, no symptoms; 1, mild symptoms (i.e., symptoms that did not affect activities of daily life); 2, moderate symptoms (i.e., symptoms that affected activities of daily life to some degree); and 3, severe symptoms (i.e., symptoms that markedly affected activities of daily life).

### Statistical analysis

We employed the Kruskal–Wallis test for sequential items in comparing three groups, followed by the Mann–Whitney *U* test adjusted by Holm’s method, and Fisher’s exact test for nominal categories, followed by the test for equal proportions adjusted by Holm’s method. We performed a multivariate linear regression analysis to compare the effects of independent variables on the degree of symptom severity. The independent variables included cancer type, age at surgery, time since surgery, BMI, menopausal status at surgery, and whether or not chemotherapy and radiotherapy were performed. We confirmed a positive correlation between age at the surgery and age at the first consultation (Pearson’s correlation coefficient *R* = 0.91) and between time elapsed since surgery and age at the first consultation (*R* = 0.45), which indicated to refrain from using age at the first consultation as a variable for further analysis. There were two patients, one with cervical and one with ovarian cancer, who had received neoadjuvant chemoradiation therapy and chemotherapy, respectively; we did not differentiate these patients in this analysis. For this analysis, patients were divided into three groups according to their age at first consultation, as linearity cannot be achieved using crude continuous values with the present small sample size: (i) < 45 years, (ii) 45–55 years, and (iii) > 55 years; the time since surgery: (i) < 1 year, (ii) 1–5 years, and (iii) > 5 years; and their BMI: (i) < 18.5 kg/m^2^, (ii) 18.5–25 kg/m^2^, and (iii) > 25 kg/m^2^. We performed a subgroup analysis with the items significantly associated with symptom severity, except for cancer type, to observe an interaction between items and the three cancer types. We used the Kruskal–Wallis test for sequential items in comparing three or more groups, followed by the Mann–Whitney *U* test adjusted by Holm’s method. All statistical analyses were conducted using R software, version 4.0.1 (The R Foundation for Statistical Computing, Vienna, Austria). We used “glm” from the package “stats” and “DAAG” for regression analysis. We used a significant level of 5% for all tests.

## Results

### Baseline characteristics of the study patients

**Fig. 1:**
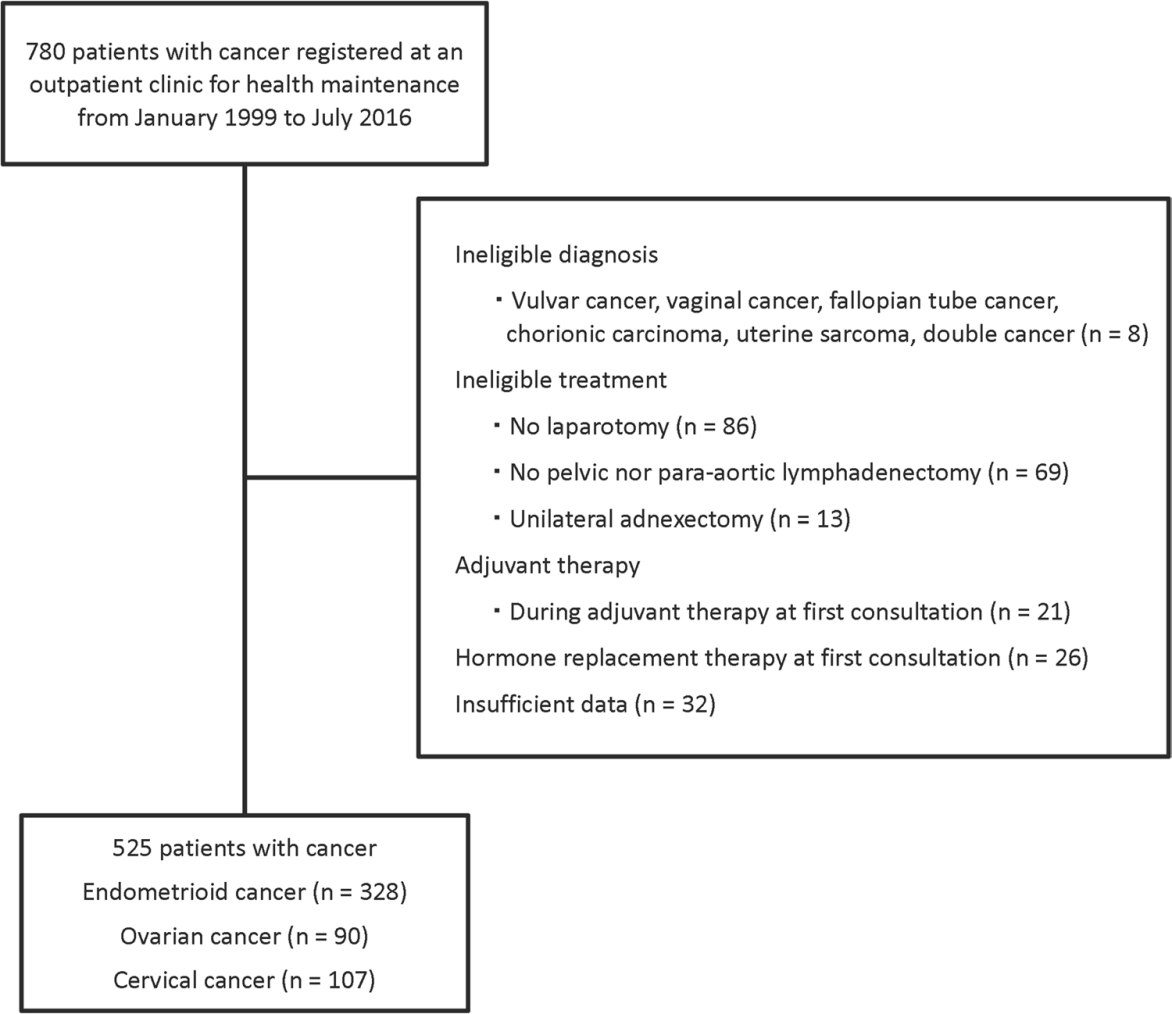
Exclusion flow chart. We initially registered 780 patients. After excluding 255 patients, we finally included 525 patients in the analysis

A total of 780 patients were examined between January 1999 and July 2016, of whom 525 met the inclusion criteria (Fig. 1). Of these 525 patients, 328, 90, and 107 had endometrial, ovarian, and cervical cancer, respectively. The baseline characteristics of patients in each of the three groups are presented in Table 1. Patients with endometrial cancer were significantly older at the first consultation and at surgery and had significantly higher BMI than patients with ovarian or cervical cancer. Among patients with cervical cancer, 26% were premenopausal at the surgery, which was a significantly higher proportion than that of patients with endometrial or ovarian cancer, as most of these patients had already undergone natural menopause. Among patients with cervical cancer, 13% had received radiotherapy post laparotomic surgery, whereas no patients with endometrial or ovarian cancer had received chemoradiotherapy as adjuvant therapy.

**Table 1:** Baseline characteristics of the study patients

Characteristics	Endometrioid cancer *N* = 328	Ovarian cancer *N* = 90	Cervical cancer *N* = 107	*p* value
Age at first consultation, years	57 (26–84)^o,c^	45.5 (30–75)^e,c^	43 (23–78)^e,o^	< 0.001
Age at surgery, years	50 (24–60)^o,c^	44 (26–56)^e,c^	40.5 (23–56)^e,o^	< 0.001
Time since surgery, months	31 (1–333)	25 (1–212)	20 (1–366)	0.343
Time since surgery, *N* (%)				
< 1 year	84 (25.6)	20 (22.2)	34 (31.8)	0.291
1–5 years	132 (40.2)	46 (51.1)	44 (41.1)	
> 5 years	112 (34.1)	24 (26.7)	29 (27.1)	
Body mass index, kg/m^2^	22.1 (12.6–38.1)^o,c^	20.5 (15.5–28.9)^e^	20.3 (13.0–30.0)^e^	< 0.001
Body mass index, *N* (%)				
< 18.5 kg/m^2^	35 (10.7)	17 (18.9)	20 (18.7)	< 0.001
18.5–25 kg/m^2^	211 (64.3)	66 (73.3)	79 (73.8)	
> 25 kg/m^2^	82 (25.0)^o,c^	7 (7.8)^e^	8 (7.5)^e^	
Menopausal status at surgery, *N* (%)				
Premenopausal	3 (0.9)^c^	2 (2.2)^c^	28 (26.2)^e,o^	< 0.001
Postmenopausal	325 (99.1)	88 (97.8)	79 (73.8)	
Additional treatment, *N* (%)				
Chemotherapy only	83 (25.3)^o^	56 (62.2)^e,c^	32 (29.9)^o^	< 0.001
Radiation only	5 (1.5)^c^	0 (0.0)	7 (6.5)^e^	
Chemoradiation	0 (0.0)^c^	0 (0.0)^c^	7 (6.5)^e,o^	
None	240 (73.2)^o,c^	34 (37.8)^e,c^	61 (57.0)^e,o^	

Data represent the median (range) unless otherwise indicated. *p* value based on Kruskal–Wallis test for sequential items followed by Mann–Whitney *U* test adjusted by Holm's method and Fisher’s exact test for nominal categories followed by the test for equal proportions adjusted by Holm’s method. Superscripts e, o, and c indicate significant difference compared with endometrial, ovarian, and cervical cancer, respectively

### Multivariate analysis on the symptom severity with baseline characteristics

Multivariate analysis of baseline characteristics revealed that two independent variables, the time elapsed since surgery and cancer type, were significantly associated with the severity of many core climacteric symptoms (Table 2). The time elapsed since surgery was significantly associated with the severities of four symptoms except for vaginal dryness, and symptom severity decreases with increasing time since surgery. The cancer type was significantly associated with three among five symptoms’ severities, namely, hot flashes, vaginal dryness, and arousal during sleep, and symptom severity increases if the cancer type is ovarian or cervical cancer. Vaginal dryness was significantly associated only with cancer type, and difficulty falling asleep was associated only with time elapsed since surgery.

**Table 2:** Multivariate analyses

	Hot flashes			Sweats			Vaginal dryness			Difficulty falling asleep			Arousal during sleep		
	Estimate	SE	*p* value	Estimate	SE	*p* value	Estimate	SE	*p* value	Estimate	SE	*p* value	Estimate	SE	*p* value
Age at surgery, years															
< 45 years	–	–	–	–	–	–	–	–	–	–	–	–	–	–	–
45–55 years	− 0.428	0.0849	< 0.001	− 0.356	0.094	< 0.001	− 0.121	0.064	0.059	− 0.037	0.095	0.696	0.04	0.090	0.658
> 55 years	− 0.404	0.180	0.025	− 0.328	0.200	0.101	− 0.171	0.136	0.208	− 0.268	0.202	0.186	− 0.064	0.192	0.741
Time elapsed since surgery, years															
< 1 year	–	–	–	–	–	–	–	–	–	–	–	–	–	–	–
1–5 years	− 0.255	0.0951	0.008	− 0.283	0.105	0.008	0.027	0.072	0.709	− 0.163	0.107	0.127	− 0.39	0.101	< 0.001
> 5 years	− 0.412	0.102	< 0.001	− 0.318	0.113	0.005	− 0.029	0.077	0.708	− 0.266	0.114	0.020	− 0.551	0.108	< 0.001
Body mass index, kg/m^2^															
< 18.5	–	–	–	–	–	–	–	–	–	–	–	–	–	–	–
18.5–25	− 0.165	0.114	0.149	0.0889	0.126	0.482	− 0.010	0.086	0.908	− 0.01	0.128	0.937	− 0.204	0.121	0.094
> 25	− 0.033	0.139	0.813	0.368	0.154	0.017	− 0.077	0.105	0.461	− 0.067	0.156	0.667	− 0.238	0.148	0.109
Menopause at surgery, yes/no	− 0.132	0.102	0.198	− 0.247	0.113	0.029	0.113	0.077	0.141	− 0.092	0.114	0.420	0.0103	0.109	0.924
Chemotherapy, yes/no	− 0.098	0.0852	0.250	− 0.004	0.094	0.970	− 0.088	0.064	0.170	− 0.053	0.096	0.582	− 0.221	0.091	0.015
Radiation, yes/no	− 0.151	0.210	0.475	− 0.124	0.233	0.596	0.104	0.158	0.512	− 0.3	0.236	0.204	0.1129	0.224	0.614
Cancer type															
Endometrial cancer	–	–	–	–	–	–	–	–	–	–	–	–	–	–	–
Ovarian cancer	0.227	0.109	0.039	0.171	0.121	0.159	0.266	0.083	0.001	0.0644	0.123	0.600	0.260	0.117	0.026
Cervical cancer	0.242	0.113	0.034	0.140	0.126	0.267	0.181	0.085	0.035	0.1941	0.127	0.127	0.350	0.120	0.004

*SE* standard error

Other variables were significantly associated with only one or two symptom severities. Symptom severity of two vasomotor symptoms, i.e., hot flashes and sweats, decreased if age was greater than 45 years at surgery. The severity of sweats increased if the patient’s BMI was more than 25 kg/m^2^ and decreased if they were menopausal at the time of surgery. The severity of arousal during sleep decreased if adjuvant chemotherapy had been performed.

**Fig. 2:**
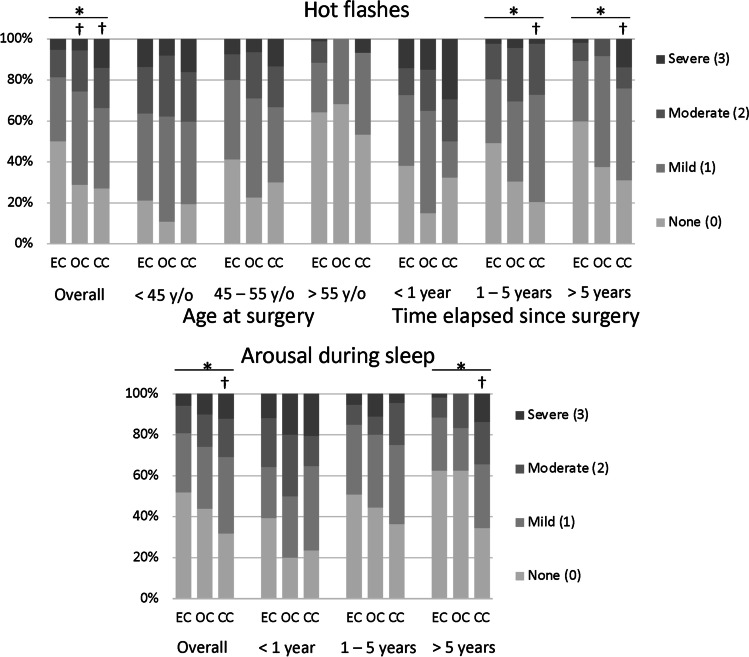
Interactions between items significantly associated with symptom severities and the three cancer types. Graphed are the severity of hot flashes and arousal during sleep along with the subgroup analysis by items significantly associated with the symptom severity via the previous regression analysis. The severity of hot flashes showed a significant difference among the three cancer types (overall); symptom severity was less in patients with endometrial cancer than in those with ovarian or cervical cancer. The significant difference among the three cancer types disappeared when the data were subgrouped by age at surgery (< 45, 45–55, and > 55 years) but remained in the subgroups of patients for whom 1–5 and > 5 years had elapsed since surgery; symptom severity was less in patients with endometrial cancer than in those with cervical cancer. The severity of arousal during sleep showed a significant difference among the three cancer types, and the symptom severity was less in patients with endometrial cancer than in those with cervical cancer (overall). We compared the severity of arousal during sleep and found that the significant difference among the three cancer types disappeared in the subgroups of patients for whom < 1 and 1–5 years had elapsed since surgery but remained for those in whom > 5 years had elapsed; symptom severity was less in patients with endometrial cancer than in those with cervical cancer. EC; endometrial cancer, OC; ovarian cancer, CC; cervical cancer, y/o; years old. *p < 0.05 by the Kruskal–Wallis test, ^†^*p* < 0.05 by the Mann–Whitney *U* test compared with endometrial cancer

Then, we focused on hot flashes and arousal during sleep with significant associations with cancer types and compared the severity of hot flashes to observe the interactions between the items significantly associated with symptom severities and the three cancer types (Fig. 2). Vaginal dryness was excluded from the analysis because only the severity of vaginal dryness was significantly associated with cancer type. Hot flashes showed significant associations with age at surgery and time elapsed since surgery along with cancer type, as revealed by the previous regression analysis. Accordingly, the severity of hot flashes showed a significant difference among the three cancer types as indicated by the Kruskal–Wallis test; symptom severity was less in patients with endometrial cancer than in those with ovarian or cervical cancer, as revealed by the Mann–Whitney *U* test. The significant difference among the three cancer types disappeared when the data were subgrouped by age at surgery (< 45, 45–55, and > 55 years) but remained in the subgroups of patients for whom 1–5 and > 5 years had elapsed since surgery, as indicated by the Kruskal–Wallis test; symptom severity was less in patients with endometrial cancer than in those with cervical cancer, as indicated by the Mann–Whitney *U* test.

Significant associations were noted between arousal during sleep and time elapsed since surgery along with cancer type. The severity of arousal during sleep was significantly different among the three cancer types, as indicated by the Kruskal–Wallis test; symptom severity was less in patients with endometrial cancer than in those with cervical cancer, as indicated by the Mann–Whitney *U* test. We compared the severity of arousal during sleep and found that the significant difference among the three cancer types disappeared in the subgroups of patients for whom < 1 and 1–5 years had elapsed since surgery but remained for those for whom > 5 years had elapsed, as indicated by the Kruskal–Wallis test; symptom severity was less in patients with endometrial cancer than in those with cervical cancer, as indicated by the Mann–Whitney *U* test.

## Discussion

In this study, we compared the severity of core climacteric symptoms after surgery and adjuvant therapy among different groups of gynecological cancer survivors. Symptoms were less severe in patients with endometrial or ovarian cancer with a longer time elapsed since surgery. Patients with cervical cancer showed greater symptom severity than those with endometrial or ovarian cancer, and their symptom severity showed no change over time. Our findings suggest the importance of climacteric symptoms in gynecological cancer survivors, and especially of cervical cancer survivors, although further studies are needed to confirm our findings.

Our results indicated that vasomotor symptoms were more severe in patients with cervical or ovarian cancer than in those with endometrial cancer, while mental symptoms, such as arousal during sleep, were more severe in those with cervical cancer than in those with endometrial cancer, possibly due to the abrupt change in sex hormone levels following bilateral ovariectomy [37]. Vasomotor symptoms were less severe with increased time following surgery and if patients were ≥ 45 years old or postmenopausal at the time of surgery. Insomnia was also improved as time elapsed following surgery. Prolonged time from surgery or a smaller change in estrogen level before and after surgery can lead to less severe symptoms. In contrast, vaginal dryness did not improve with time elapsed since surgery because this symptom occurs due to a low estrogen level itself that do not improve as time elapsed since surgery [38]. Furthermore, symptom severity in patients with cervical cancer showed no change over time compared with that in patients with ovarian cancer, although both these groups of patients had undergone bilateral ovariectomy at similar ages. This result suggests the involvement of other factors that might have affected symptom severity along with a decrease in estrogen levels.

Mood and mental health are significantly associated with factors, such as education, income, and the presence or absence of a partner [19]. Numerous studies have compared climacteric symptoms after cancer treatment and adjuvant therapy across different socioeconomic strata [20], and socioeconomic background can present risks for menopausal symptoms [39]. However, we did not investigate differences in the socioeconomic background of patients. In Japan, it is often difficult to ask patients their socioeconomic status. Moreover, there are differences between Japan and other countries with respect to healthcare information and social background. Thus, more studies are required to clarify the contribution of these currently unmeasured factors, which might be represented by cancer type.

This study had certain limitations. First, it was performed at a single institution. Second, there was no control group without a cancer diagnosis, and we could not compare the symptom severity with those without gynecological cancer. Third, we did not classify patients with respect to cancer stage. Although several reports have addressed the relationship between cancer stage and the QOL of patients with gynecological cancer, their results are inconsistent [40–42] and the relationship between cancer stage and the climacteric symptoms is unknown. Therefore, further studies are needed to address this issue. Fourth, some of the patients in this study were enrolled more than 20 years before, and surgical advances in the past two decades may have resulted in improvements in postoperative symptoms [43]. Several procedures might affect post-surgical complications (e.g., simple (sometimes extended), semi-radical, and radical hysterectomy). Surgical procedures have improved significantly, and several patients underwent surgery at another institution, making it difficult to obtain information concerning the performed procedure. Thus, we decided that it was difficult to use procedures as an independent variable. Finally, this study was cross-sectional; information was obtained only from one questionnaire at completely random time points since surgery. The results may have differed with other time points of assessment. Thus, changes in each patient’s symptoms during the study are unknown, and it is unclear whether these patients had climacteric complaints before their oncological surgery/therapy or only after surgery, even though we have information of participants’ menopausal state at the surgery. In addition, the sample size was set based on the number of best available cases over the study period, and no sample size calculation was performed. The observed non-significant results in the present analysis may be attributed to beta error by smaller numbers with stratification.

## Conclusions

Patients with cervical cancer showed more severe climacteric symptoms than those with endometrial or ovarian cancer. Overall, the symptoms were less severe in patients with longer time elapsed since surgery. However, in patients with cervical cancer, the core symptom severity did not change significantly with the time elapsed since surgery. Patients with cervical cancer may require more prompt intervention, including symptomatic treatment and longer follow-up period, than those with endometrial or ovarian cancer.

## Supplementary Information

Below is the link to the electronic supplementary material.

Supplementary file1 (XLSX 12 KB)

## Data Availability

The datasets used and/or analyzed during the current study are available from the corresponding author on reasonable request.

## Abbreviations

CC: Cervical cancer
EC: Endometrial cancer
OC: Ovarian cancer
QOL: Quality of life
y/o: Years old
